# Kinetic barriers in the isomerization of substituted ureas: implications for computer-aided drug design

**DOI:** 10.1007/s10822-016-9913-4

**Published:** 2016-06-07

**Authors:** Johannes R. Loeffler, Emanuel S. R. Ehmki, Julian E. Fuchs, Klaus R. Liedl

**Affiliations:** Institute of General, Inorganic and Theoretical Chemistry, Faculty of Chemistry and Pharmacy, University of Innsbruck, Innrain 82, 6020 Innsbruck, Austria

**Keywords:** Cis/trans isomerization, Starting structure, Matched molecular pairs, Thermodynamic integration, Umbrella sampling, Potential of mean force, Drug design, Molecular modeling, Urea

## Abstract

Urea derivatives are ubiquitously found in many chemical disciplines. *N*,*N*′-substituted ureas may show different conformational preferences depending on their substitution pattern. The high energetic barrier for isomerization of the cis and trans state poses additional challenges on computational simulation techniques aiming at a reproduction of the biological properties of urea derivatives. Herein, we investigate energetics of urea conformations and their interconversion using a broad spectrum of methodologies ranging from data mining, via quantum chemistry to molecular dynamics simulation and free energy calculations. We find that the inversion of urea conformations is inherently slow and beyond the time scale of typical simulation protocols. Therefore, extra care needs to be taken by computational chemists to work with appropriate model systems. We find that both knowledge-driven approaches as well as physics-based methods may guide molecular modelers towards accurate starting structures for expensive calculations to ensure that conformations of urea derivatives are modeled as adequately as possible.

**Electronic supplementary material:**

The online version of this article (doi:10.1007/s10822-016-9913-4) contains supplementary material, which is available to authorized users.

## Introduction

**Fig. 1:**
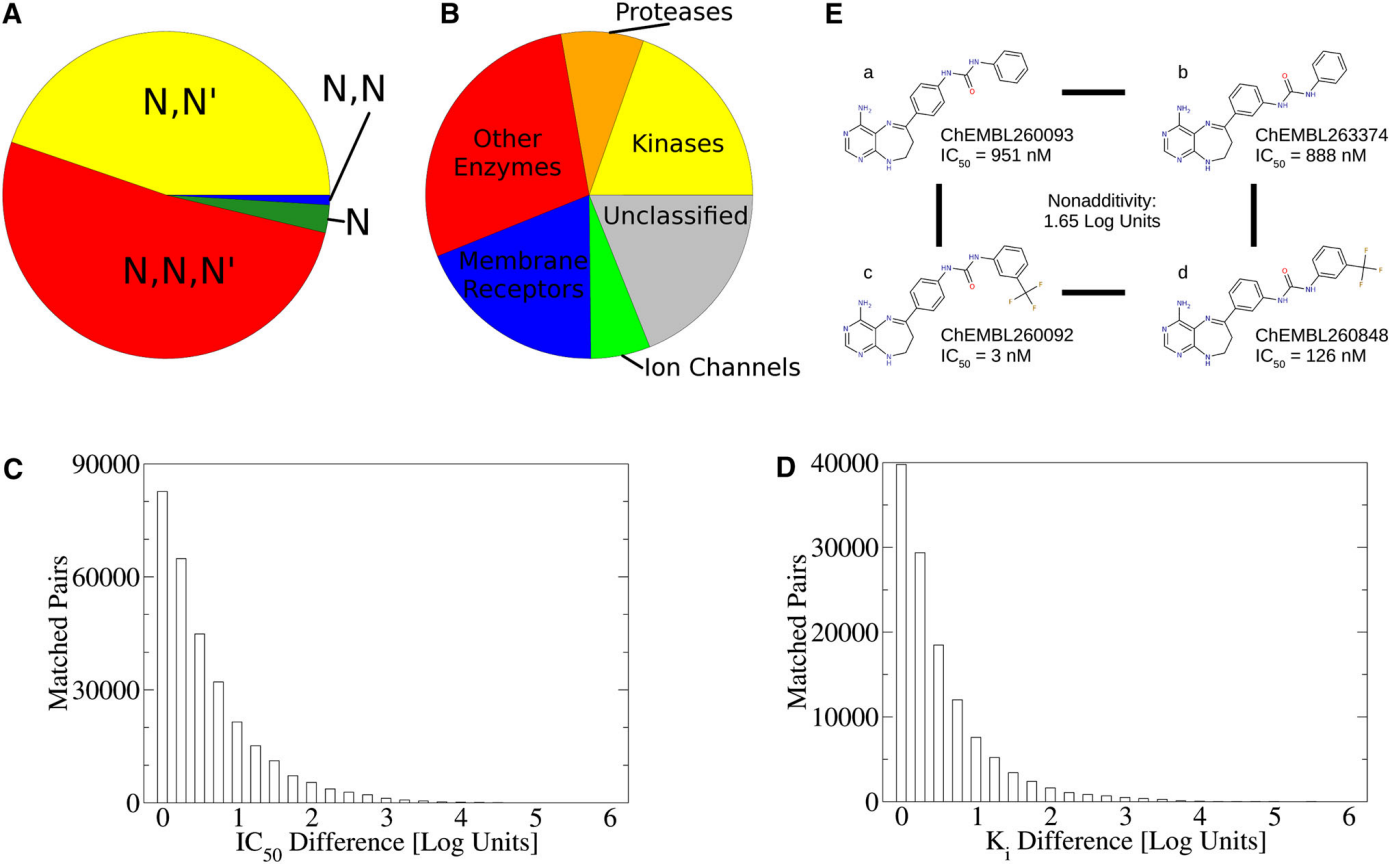
Bioactivities of urea derivatives: **a** The vast majority of urea-derived compounds in ChEMBL is either *N*,*N*′-di-substituted (*yellow*) or tri-substituted (*red*). Mono-substituted ureas (*green*) as well as *N*,*N*-di-substituted ureas (*blue*) are less frequent. **b** Urea derivatives are known to exhibit a variety of bioactivities targeting kinases (*yellow*), proteases (*orange*) and other enzymes (*red*). Furthermore, membrane receptors (*blue*) and ion channels (*green*) are known targets as well as further unclassified targets (*grey*). **c**, **d** Affinity differences (**c**: IC_50_ and **d**: K_i_ data) derived from matched pairs among urea derivatives indicate that substitutions of urea compounds may lead to major changes in binding potency. **e** An example double transformation cycle of VEGFR2 inhibitors (with ChEMBL compounds IDs and activity data from [11]): A change in linker substitution between compounds a and b (para to meta) leads to little change in binding affinity. Strinkingly, compound a receives a major affinity boost of 2.5 log units by trifluouromethyl substitution (compound c). The effect of the identical substitution from compound b–d shows a much smaller gain in affinity, leading to a non-additivity of 1.65 log units

Urea derivatives are broadly used in various disciplines of chemistry including catalysis, metal binding and supramolecular chemistry [1]. Furthermore, urea substructures are prominent in drug design and medicinal chemistry, where they are introduced to allow strong hydrogen bonding [2] which might lead to cooperative effects [3]. Additionally, many bioisosteres for urea have been developed and were successfully applied in structure-based design campaigns [4]. Drugbank (version 4.3 [5]) lists 148 acyclic urea-derived compounds among approved and investigational drugs including prominent examples like ritonavir, sorafenib, or regorafenib. The bioactivity database ChEMBL (version 20 [6]) lists 76,494 urea-derived biologically active molecules, thus corresponding to more than 5 % of indexed molecules in total. Thereof, 34,232 *N*,*N*′-di-substituted and 39,426 tri-substituted ureas form the major contributors (see Fig. 1a). Interestingly, terminal urea groups are rarely found contributing only 2092 as mono-substituted ureas and 743 compounds as *N*,*N*-di-substituted ureas in addition to the parent compound urea itself. No tetra-substituted ureas are listed in ChEMBL at all.

These compounds are distributed over all major target classes in ChEMBL (see Fig. 1b). Aryl-urea substructures are for example used to target the inactive DFG-out conformation in type II kinase inhibitors [7]. Such compounds allow unique specificity profiles amongst kinases since a hydrophobic region apart from the conserved ATP pocket is targeted [8]. Matched molecular pairs, compounds differing in a single chemical modification [9], amongst urea-derived compounds vary in binding potency over several orders of magnitude up to six log units (see Fig. 1c, d). Additionally, double transformation cycles extracted as cycles of four matched pairs [10] indicate non-additivity of substituent contributions to binding free energy. These non-additive contributions to binding free energy likely arise due to changes in ligand conformation and/or binding pose (see Fig. 1e for an example).

Computational strategies to estimate differences in free energy of binding to biological receptors are emerging as key tools in rational drug design [12]. Though force field inadequacies and limited sampling times inherently limit accuracy, free energy calculations provide a valuable source of directions for lead optimization [13]. Special care needs to be taken to accurately represent the system in computationally demanding free energy calculations both on receptor and ligand side [14, 15].

Urea derivatives pose additional challenges on the computational chemist, since the planar systems may adopt different conformations. In general, the trans state is preferred for ureas, esters and amides [16]. This effect stems from increased steric repulsion of substituent groups in cis state [17]. *N*,*N*′-diethyl-urea is found in trans/trans conformation in apolar media and may give rise to self assembly via intermolecular hydrogen bonding [18]. Knowledge-driven analysis of torsion profiles from small molecule crystal structures also revealed a strong preference for the trans state over cis with a tolerance of only 20° [19]. The absolute minimum energy conformation of substituted ureas might in fact deviate slightly from planarity [20]. Major changes of logD and solubility upon N-methylation have recently been described for urea derivatives and been attributed to conformational transitions [21] which appears reasonable since urea-derived compounds may readily form intramolecular hydrogen bonds and thereby alter physico-chemical properties [22].

**Fig. 2:**
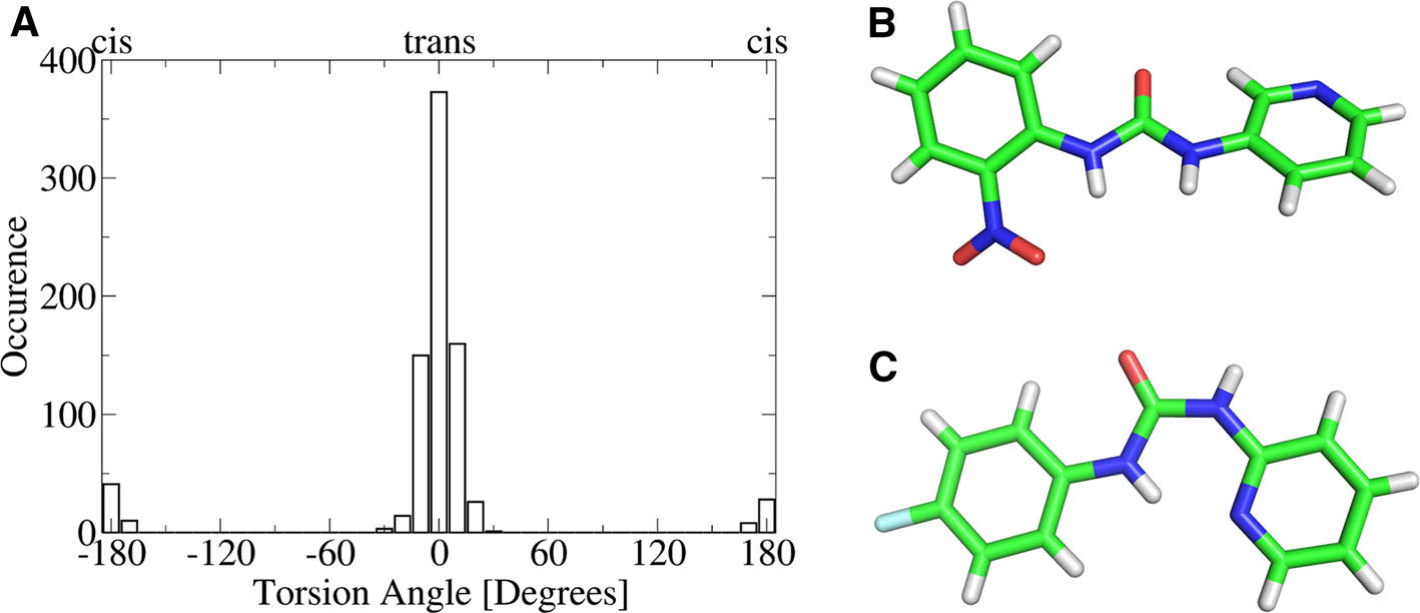
Urea conformations present in the CSD: **a** A histogram of occurring 814 torsion angles in acyclic *N*,*N*′-disubstituted ureas reveals a clear preference for trans states over cis states. **b** 1-(2-nitrophenyl)-3-pyridin-3-ylurea (CSD: WOMHUD) shows a planar trans/trans conformation. **c** By contrast, 1-(4-fluorophenyl)-3-pyridin-2-ylurea adopts a cis/trans conformation (CSD: WOMGUC) that is stabilized via an intramolecular hydrogen bond between urea and pyridine

To characterize conformational preferences of urea substructures we mined the Cambridge Structural Database (CSD) and extracted conformations for 407 indexed *N*,*N*′-disubstituted ureas with determined three-dimensional structure. Two torsions per molecule around the urea substructure were analyzed and yielded 814 torsion angles (see Fig. 2a). 716 of those (88 %) were found to lie within 0° ± 20°, representing the dominant planar trans conformation. 1 % of torsions are found between 20° and 30° and deviate from the trans state slightly. 11 % of urea compounds are found in planar cis conformation (±180° ± 20°), whereas all other ranges of torsion angles are not populated. 320 structures (79 %) are found in the trans/trans state with both torsion angles within 0° ± 30°. 87 molecules (21 %) are found with one torsion in cis and one in trans (cis/trans state), whereas not a single molecule in CSD is found in cis/cis state. Intramolecular hydrogen bonding is found to trigger conformational changes between the trans/trans and cis/trans state (see Fig. 2b, c for an example).

**Fig. 3:**
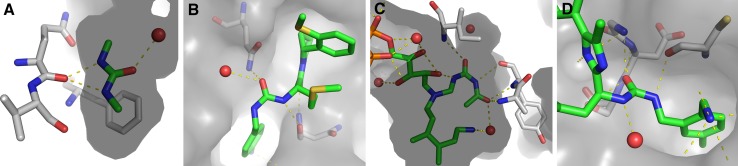
Representative urea conformations extracted from the PDB: Ligands and protein interface residues are shown in stick representation in elemental colors (carbon: *white* in proteins, *green* in ligands), additionally the protein Van der Waals surface is shown in *grey*. Water molecules are shown as *red* sphere, polar contacts with a distance smaller than 3.3 Å are shown as *yellow dashed lines*. **a** The co-crystal structure of a bacterial urea transporter and *N*,*N*′-dimethyl-urea shows the low energy trans/trans conformation that is stabilized via hydrogen bonds to a protein backbone carbonyl and to a water (PDB: 3K3G [24]). **b** The binding site shape of peptidyl-prolyl cis–trans isomerase together with the local hydrogen bonding partners enforce a cis/trans conformation in the urea substructure of a bound ligand (PDB: 4ZSD [25]). **c** A substrate analogon shows a rare cis/cis urea conformation when bound to a bacterial 6-hydroxy-l-nicotine oxidase (PDB: 3NN6 [26]). **d** An unlikely non-planar conformation is represented in the PDB in a co-crystal structure of factor XIa and a urea-based inhibitor (PDB: 4X6M [28])

Similar trends were observed when querying the Protein Data Bank (PDB) [23] for urea derivatives. We retrieved a manually curated data set of 120 structures, some of which having multiple ligands bound or multiple conformations modeled. In agreement with CSD data, the majority of ligands (93 %) is found in trans/trans conformation, whereas only few adopt a cis/trans state (see Fig. 3 for examples). One single structure is even found in cis/cis conformation. Here, the cis/cis conformation of the urea is implied both by pocket shape as well as local hydrogen bonding patterns and can therefore be explained as a result of ligand strain [27]. Solvent-exposed urea motifs are predominantely found in trans/trans state, while the cis/trans state can be readily implied by protein-ligand interactions. The PDB also contains rare urea conformations as deposited for urea-linked factor XIa inhibitors bearing alternative P1 groups [28]. Whether the observed non-planar substitution pattern (torsion angle: −137.7°) represents reality or an experimental artifact remains elusive.

The factual absence of structures showing other torsion angles involving the urea substructure than either the dominant trans conformation or the minor cis conformation implies a high energy barrier for the involved torsions. Herein we investigated whether state-of-the-art simulation techniques provide a long enough time scale to sample transitions between those separated energetic minima. We found that unbiased simulation protocols are insufficient to sample the involved transitions and therefore extra care needs to be taken by molecular modelers to appropriately represent conformations of substituted ureas in their simulation setups.

## Methods

### Data mining

We used the web interface of ChEMBL to extract compounds with urea substructure from ChEMBL20 [6]. Subsequently, we searched for matched molecular pairs amongst the urea compounds using the search algorithm of Hussain and Rea [29] as implemented in RDKit [30]. Presented matched molecular pairs show replacements directly connected to the urea fragment and fulfill previously published quality criteria [31]. K_i_ and IC_50_ data were treated separately in the extraction of matched pairs and are based on the identical source publication and assay identifier to minimize experimental uncertainty. Creation of double transformation cycles based on urea-containing matched pairs was performed as described earlier [10].

We screened the CSD small molecule database (version 5.36, November 2014) using ConQuest 1.17 [32]. We retrieved structures of acyclic *N*,*N*′-di-substituted ureas and calculated dihedral angles over both sides of the urea fragment including the substituents’ first atoms. Our search space was defined as organic molecules with available 3D structure, no disorder and R-factor ≤0.05.

Similarly, we used the PDB web interface to search for *N*,*N*′-di-substituted ureas amongst protein-ligand complexes. Since a limitation to acyclic ureas only was impossible, we performed a manual cleaning step to remove ligand bearing the urea fragment within a cyclic substructure. Thereby, the majority of hit structures were discarded, e.g. due to presence of biotin derivatives. Molecular structures were visualized using PyMOL (version 1.6.0.0, Schrodinger LLC, 2013).

### Molecular dynamics simulations

We performed molecular dynamics simulations of *N*,*N*′-dimethyl-urea to investigate kinetic accessibility to cis/trans isomerization. Therefore, we parametrized the ligand using the Generalized Amber Force Field (GAFF) [33] and AM1-BCC charges [34] as implemented in Amber14 [35]. We followed the suggested work-flow for non-standard residues in Amber using antechamber for setting of GAFF atom types and parameter assignment. Planarity was therefore enforced with a general improper torsion term as applied for esters or amides.

We investigated three starting conformation: *trans*/*trans*, *cis*/*trans* and *cis*/*cisN*,*N*′-dimethyl-urea. Subsequent to energy minimization, temperature (300 K) and pressure (1 bar) equilibration, all systems were sampled for 25 ns in explicit TIP3P water environment [36] using the GPU implementation of pmemd [37]. Systems were analyzed by extraction of dihedrals along the SMARTS pattern CNC=O as well as hydrogen bond counting applying default cut-offs in cpptraj (maximum heavy atom distance: 3.0 Å, minimum angle of interacting atoms: 135°) [38].

To enforce transitions between the cis and trans energy minima we additionally performed umbrella sampling simulations. Thereby, the dihedral angle over the urea substructure was slowly biased by a harmonic extra potential from cis to trans state and vice versa. Simulations were performed both in solution and vacuum in 121 windows with a shift of 3° in the dihedral minimum. The slope of the harmonic potential was set to 200 kcal/mol over 180°. Each window was sampled over 500 ps and the resulting distribution was stored in 10,000 snapshots. Subsequently, the distribution of states was re-weighted using the weighted histogram analysis method (WHAM) including dihedral periodicity [39]. Error bars were derived from ten-fold Monte Carlo random subset sampling.

We performed thermodynamic integration (TI) calculations to investigate free energy changes in a protein-ligand system caused by inversion of a urea substructure. Therefore, we protonated the crystal structure of vascular endothelial growth factor receptor 2 (VEGFR-2) tyrosine kinase in complex with a benzamidiazole urea inhibitor (PDB: 2OH4 [40]) for simulations using protonate3D [41]. The system was parametrized, solvated and energy minimized using the Amber14 package as described above using Amber ff99SB-ILDN for protein atoms [42]. After an NpT equilibration over 200 ns we transformed the trifluoromethyl group (CF_3_) close to the ligand’s urea substructure (a topology similar to regorafenib) to a thio-trifluoromethyl group (SCF_3_) using a one step TI approach using soft-core potentials [43]. The transformation was conducted using 22 λ-windows with 1 ns sampling time each. Error bars for free energies were extracted from ten-fold trajectory splitting. The template ligand (CF_3_) was simulated in trans/trans configuration whereas the target ligand (SCF_3_) was simulated in both trans/trans and cis/trans configuration. To generate the starting structure the torsion angle over the terminal aromatic ring was adjusted manually to cis/trans state (see Supporting figure 1 for graphical representations). Additionally, we performed a simulation of the inversion of the template ligand (CF_3_) in solution from trans/trans to cis/trans and vice versa.

### Quantum mechanical calculations

We conducted dihedral scans at HF/6-311G level for three compounds using Gaussian03 [44]. First we re-examined the torsion of *N*,*N*′-dimethyl-urea at quantum mechanical level and added a cyclophilin D ligand (1-(4-aminobenzyl)-3-[(2S)-4-(methylsulfanyl)-1-{(2R)-2-[2-(methylsulfanyl)phenyl]pyrrolidin-1-yl}-1-oxobutan-2-yl]urea, PDB ligand ID: 7I6) along with the VEGFR-2 ligand used for TI calculations as real life examples. Rotational scans were performed on the dihedral angle over the CNC=O SMARTS pattern which was increased in 5° and 10° steps respectively from 0 (trans state) to 180° (cis state). For ligand 7I6 the torsion profile for the 4-amino-benzyl substituted amide nitrogen was recorded, for the VEGFR-2 ligand we profiled the torsion for the 3-trifluoromethyl-phenyl amide nitrogen. *N*,*N*′-dimethyl-urea was scanned twice, with and without a constraint enforcing a planar conformation of the nitrogen amides in order to bypass potential changes in hybridization state. To counteract hysteresis effects from the torsion scan, we subsequently energy minimized identified minimum and transition state structures for the *N*,*N*′-dimethyl-urea system. Additionally, we performed a torsion scan for all three systems using the GAFF parameters derived for molecular dynamics simulations.

## Results

**Fig. 4:**
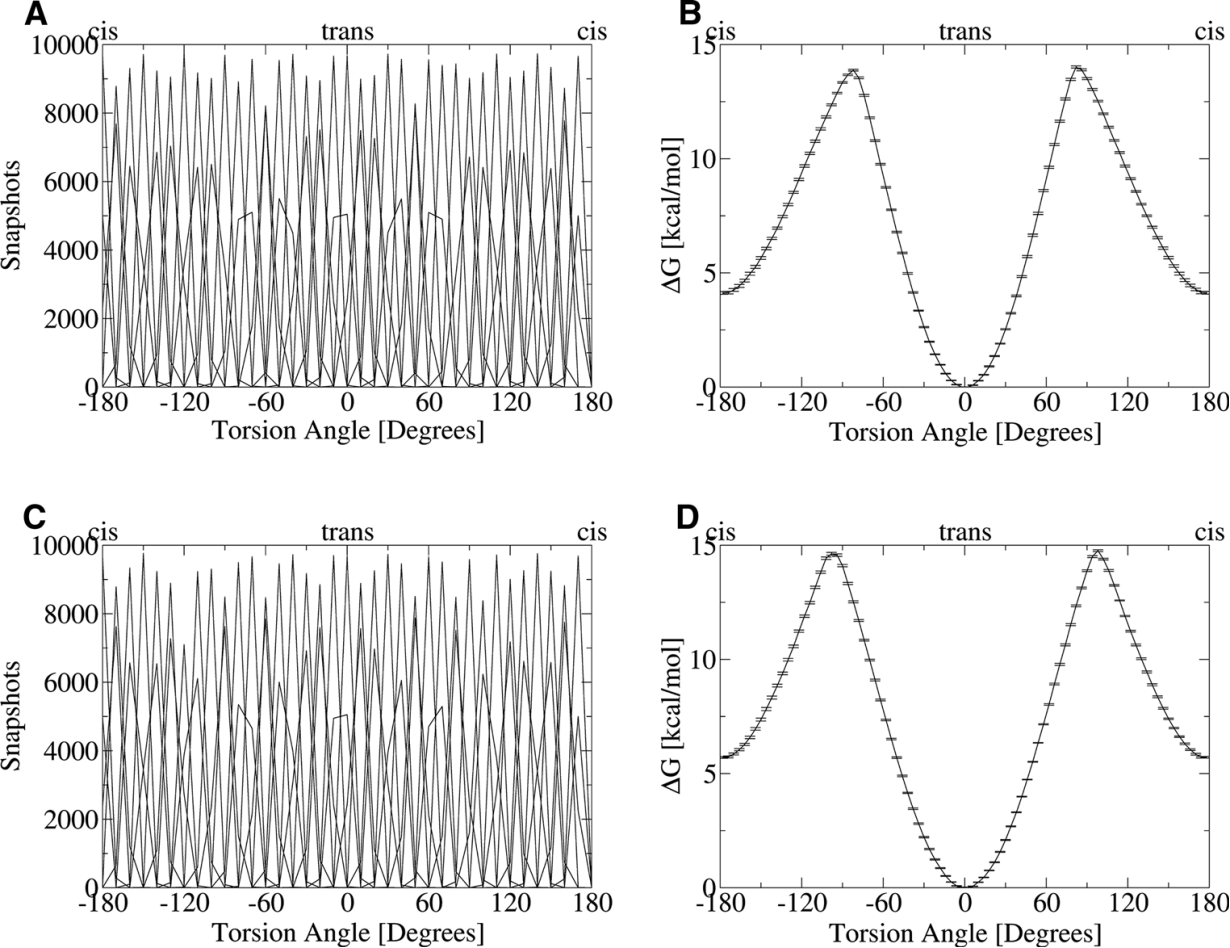
Umbrella sampling simulations on *N*,*N*′-dimethyl-urea: **a** State distributions gathered from umbrella sampling simulation in vacuum shown as a histogram with 10° bin width. **b** Reconstructed free energy profile for the urea inversion from the umbrella sampling in vacuum. The trans configuration (0°) is the global energy minimum, while the cis state (±180°) is only a local minimum with an energy offset of ΔΔG_Vacuum_ = 4.1 kcal/mol. The transitions state is observed at ±82° and a relative energy of ΔΔG_Vacuum_ = 14.0 kcal/mol. **c** State distributions extracted from umbrella sampling in explicit solvation. **d** The reconstructed free energy profile in explicit solvation is similar to the corresponding profile in vacuum. Shifts in energy differences in comparison to the simulations in vacuum are observed between cis and trans state (ΔΔG_Solvation_ = 5.7 kcal/mol) as well as between trans state and transition state (ΔΔG_Solvation_ = 14.8 kcal/mol). Additionally, the transition state is shifted to ±98° in explicit solvation

We performed molecular dynamics simulations of *N*,*N*′-dimethyl-urea in three conformational states (trans/trans, cis/trans, cis/cis). We did not observe a single conformational transition from cis to trans or vice versa over a sampling time of 25 ns in explicit solvation. To enforce the inversion of the urea conformation we added biasing potentials to our simulations by performing an umbrella sampling. Simulations were performed in vacuum as well as explicit solvation and both lead to similar free energy profiles (see Fig. 4). The trans state is identified as global energy minimum, whilst the cis state is a local energy minimum with an intrinsic strain energy of 4.1 kcal/mol in vacuum and 5.7 kcal/mol in explicit solvation. Error bars from simulations are found well below 0.1 kcal/mol and strengthen confidence in the presented free energy profiles. The barrier height for inversion from the trans to cis state is found as high as 14.0 kcal/mol in vacuum and 14.8 kcal/mol in solution.

Since both energy differences from trans state to cis state as well as to the transition state are elevated in solution compared to the simulations in vacuum, the trans state appears additionally stabilized by surrounding water molecules. We therefore analyzed hydrogen bonding patterns of the three different urea conformations occurring in the 25 ns long unbiased simulations. We found that *trans*/*transN*,*N*′-dimethyl-urea shows the strongest hydrogen bonding network with surrounding water molecules. Thereby, the carbonyl function on average forms 1.69 hydrogen bonds, whereas the amide nitrogens donate 0.88 hydrogen bonds to the solvation shell (total 2.57). Inversion of one bond torsion to the cis/trans state reduces hydrogen bonds to 1.60 for the carbonyl and 0.57 for the amide nitrogens (total 2.17). A slight increase in hydrogen bonds is observed for the cis/cis state of *N*,*N*′-dimethyl-urea, where on average 1.51 hydrogen bonds are formed from the carbonyl and 0.72 from the amide nitrogens (total 2.22). Therefore, the trans/trans state appears stabilized in comparison to other conformations not only by its internal conformational energy but also via gains in hydrogen bonding to the solvation shells.

**Fig. 5:**
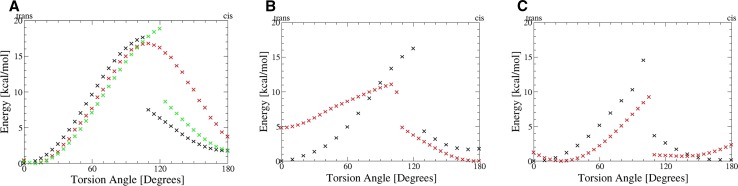
Torsional scans at HF/6-311G level and using GAFF: **a** The torsion angle around the O=CNC SMARTS pattern in *N*,*N*′-dimethyl-urea was varied from trans state (0°) to cis state (180°) without further constraints (*black*) and enforcing a planar amide conformation (*red*). The energy minimum is observed around 10° and is thus slightly non-planar when enforcing amide planarity, whilst the cis state (180°) is found at 3.7 kcal/mol elevated energy. The energy barrier between both states is identified at 110° and 16.8 kcal/mol. An unconstrained scan leads to a lower energy for the cis state but a similar barrier height. GAFF is found to reproduce the quantum mechanics-derived energy profile very well (*green*). **b** Varying the same torsion angle in the urea substructure of ligand 7I6 leads to a similar torsion profile (*black*). The global energy minimum is found in trans state whilst the cis state is a local energy minimum with an energy offset of +1.7 kcal/mol at 170°. The barrier between both states is found at 120° and 16.2 kcal/mol. The energy profile derived from GAFF (*red*) shows major disagreement with quantum mechanics since a conformational change in the ligand is observed at the beginning of the scan. **c** Energy profile for the torsion scan of the VEGFR-2 ligand: We find an energetic barrier of 14.5 kcal/mol (100°) separating the global energy minimum in trans state from the cis state (+0.15 kcal/mol). The GAFF-derived profile (*red*) shows agreement around the energy minima but clearly underestimates the barrier height in this case

To assess the accuracy of force field-based molecular mechanics based simulations, we performed dihedral scans of *N*,*N*′-dimethyl-urea, cyclophilin D ligand 7I6 and a VEGFR-2 ligand at HF/6-311G level and compared them to GAFF energies (see Fig. 5). We found similar energy profiles for torsional modifications as extracted from simulation data. Within the constrained dihedral scan the lowest energy for *N*,*N*′-dimethyl-urea was found for a slightly non-planar conformation at 10°. The cis state is identified as local minimum with an energy difference of +3.7 kcal/mol. The barrier height between both states is found to be 16.8 kcal/mol at 110°. If no constraint is used, a hysteresis effect is observed in the energy profile until the planarity of the urea fragment is re-established at 130° subsequent to a drop in energy. The energy barrier crossed at 120° is found to be 15.9 kcal/mol and the energy difference of trans and cis state is significantly lower compared to the constrained scan (+1.1 kcal/mol). Energy minimizations were performed for lowest energy structures from torsion scans and resulted in a minimum for the trans state at 9° compared to 159° for the cis state. The observed energy difference between both states was found to be 1.5 kcal/mol. The barrier was located at 119° and at 15.3 kcal/mol after zero point energy correction. Energy profiles derived from GAFF showed similar trends as quantum mechanics-derived profiles. We find the cis state energetically less favored and to be separated from the trans state by an energy barrier of 18.8 kcal/mol at 120°.

Ligand 7I6, representing a real drug design example, shows a similar energy profile along the urea torsion as the model system *N*,*N*′-dimethyl-urea. The global minimum is found in trans state (0°), whereas the cis state is 1.7 kcal/mol higher in energy at 170°. The energetic barrier separating both states is found to reach 16.2 kcal/mol at 120° in this case. The VEGFR-2 ligand shows a comparable barrier height and location with 14.5 kcal/mol at 100°. Still, we find only little energy difference between the global minimum at the trans state and the cis state (+0.15 kcal/mol). For both real world examples we observe stronger deviations of the GAFF energy profiles versus the quantum mechanics-derived profiles. Energy barriers are significantly underestimated and relative energy levels of cis and trans states are shifted in case of cyclophilin D ligand 7I6.

We performed TI simulations to investigate free energy differences between urea conformational states in a protein-ligand system. Therefore, we kept the template VEGFR2 ligand in trans/trans configuration as resolved in the crystal structure and transformed it to the target ligand in both trans/trans and cis/trans state. We found major differences in resulting free energy profiles (see Table 1). A transformation of the co-crystallized CF_3_-substituted ligand to the SCF_3_-substituted target compound yields a difference free energy of the free energy of binding of −0.84 kcal/mol when both ligand are simulated in trans/trans state (see Supporting Figure 2 for all TI free energy profiles). Assuming a switch of the target ligand conformation to cis/trans would alter the free energy difference to +20.81 kcal/mol, thus indicating strong repulsion of the ligand. The situation is even worse when comparing across conformational states of the target ligand. Binding free energy differences of +80 to +110 kcal/mol are obtained when comparing those different states of the ligand. When inverting the urea conformation of the template ligand (CF_3_) in free solution we observe a free energy penalty of 2.80 kcal/mol from trans/trans to cis/trans and −4.12 kcal/mol for the opposite direction. These values clearly demonstrate that the trans/trans state is the lowest energy conformation of our model ligand.

**Table 1:** Free energy differences from TI simulations: depending on the conformational state of the target ligand different free energy differences are recovered

Template	Target	Conformation 1	Conformation 2	Environment	ΔG (kcal/mol)	Error (kcal/mol)
CF_3_	SCF_3_	Trans/trans	Trans/trans	Solvent	87.93	0.11
CF_3_	SCF_3_	Trans/trans	Trans/trans	Protein	87.09	0.24
CF_3_	SCF_3_	Trans/trans	Cis/trans	Solvent	−21.43	0.58
CF_3_	SCF_3_	Trans/trans	Cis/trans	Protein	−0.62	3.59
CF_3_	CF_3_	Trans/trans	Cis/trans	Solvent	2.80	0.95
CF_3_	CF_3_	Cis/trans	Trans/trans	Solvent	−4.12	0.89

Error bars for predictions increase when the ligand conformation is switched, especially in presence of the protein

## Discussion

In agreement with literature data we have demonstrated that urea substructures give rise to distinct conformational states at room temperature. The lowest energy conformation for most molecules is the trans/trans state. Still, also alternative conformational states of urea fragments are thermally accessible and thus need to be considered in high quality modeling approaches. These higher energy states are the cis/trans state and the cis/cis conformation, where the latter state suffers from additional syn repulsion of methyl groups. Using TI simulations we showed that calculated free energy differences depend drastically on the ligand setup. Whilst we observe perfect agreement with experiment when comparing ligands in trans/trans state (ΔΔG_calculated_ = −0.84 kcal/mol versus ΔΔG_experiment_ = −0.80 kcal/mol [40]), we observe large and thermodynamically unreasonable deviations of the calculated binding free energies when assuming a cis/trans state for the target ligand. Thus, a correct ligand starting conformation is of utmost importance for the accuracy of TI calculations although the correct conformational state might not be immediately obvious in case of urea-derived compounds. Here, molecular dynamics simulations seeded with an ensemble of different starting conformations might allow to identify the conformation most suitable for receptor binding [45].

The three conformational states of urea derivatives are separated by high energetic barriers that we estimated in the range of 14–16 kcal/mol. This finding is in agreement with experimental data for the solid state, where a barrier of 18.5 kcal/mol has been reported [46]. Given the additional strong hydrogen bonding network in solid state, a reduction of the barrier height in solution and vacuum has to be expected. The isomerization of urea fragments in small molecules is therefore inherently slow with half lives in the area of milliseconds to seconds. These time scales are not accessible by state-of-the-art molecular dynamics simulation approaches that are limited to microsecond dynamics in proteins [47]. One might therefore consider urea substructures atropisomers, and thus separate compounds, on the current simulation time scale. Although in general a rotational barrier of 20–30 kcal/mol is accepted for real world atropisomerism [48, 49], microsecond molecular dynamics simulation will typically not allow to cross barriers of 15 kcal/mol.

Real world atropisomers include well-known biaryls as well as further chemical classes including *N*,*N*′-diarylureas [50]. Here, it is evident that substitution patterns at the urea scaffold play a major role in determining lowest energy conformations as well as barrier height. Aromatic substitution on ureas represent special cases that may give rise to intramolecular stacking interactions and thereby govern three-dimensional orientation of substituents [51, 52]. Cis/trans conformational switching of aromatic substituted ureas has been observed upon simple change of solvent properties or methylation [53]. Usually, stacked conformations (cis/cis) dominate for simple aromatic ureas as shown by combination of X-ray crystallography, NMR and extensive calculations [54]. Interestingly, our analysis of PDB structures did not reveal a single collapsed di-aryl-urea structure. Thus, one might speculate that either compounds with this conformational preference are in general disfavored in protein binding or adopt a structure with larger accessible surface area upon binding. For accurate modelling of flexible ligand systems the energetic offset (ligand strain) between unbound conformation and receptor-bound conformation needs to be included. Sufficient conformational sampling of the ligand in unbound state can help to unravel the pre-existing population of the bound state, and thus its free energy difference, if a mechanism of conformational selection applies to the studied system [55].

Beyond the class of urea-containing compounds, benzoylureas have been described as removable inducers of cis amides in the synthesis of cyclic amides as precursors of macrocycles and peptidomimetics [56]. The cis/trans state favored in benzoylureas has also been observed in urea-bearing peptidomimetics in solution [57]. Additionally, cis/trans isomerization is a crucial parameter in protein environments, where only 0.03% of amides show a cis conformation [58] with only a few of them not involving proline residues [59]. The energy difference between cis and trans amide is about +2.8 kcal/mol and brings the need for extra hydrogen bonding to stabilize the cis conformation [60]. Prolyl isomerases catalyze the crucial isomerization step in protein folding that in some cases can even be rate limiting for the whole folding process [61]. Amide isomerization rates in model peptides have been shown to reach millisecond to second time scales [62] and thus will not be accessible with typical simulation approaches just like inversions of urea conformations.

Modeling therefore requires special attention for correct system setup not only for the ligand but also for the protein side whenever internal hindered rotations are involved. Potential quality issues in starting structures (e.g. urea conformations strongly deviating from planarity as discussed earlier) might therefore hamper precise molecular modeling. Usage of high quality data sets [63] and critical assessment of starting conformations by experienced modelers are therefore key to successful predictions. Additionally, quantum-mechanical calculations as well as data mining in crystallographic databases might be considered helpful in identifying limitations in single starting configurations. In general, urea derived compounds and similar classes like thioureas, carbamates, thiocarbamates and amides require special attention for high quality molecular modeling.

## Conclusion

Using a combination of data mining, quantum mechanical calculations and molecular simulations techniques we showed that the properties of urea substructures pose significant challenges on the molecular modeler. As both trans/trans and cis/trans conformation appear frequently in protein-ligand complexes, accurate modeling might require exploration of both conformers independently since the energy barrier for inversion is too high to be sampled using state-of-the-art simulation time scales. An attractive alternative could be the application of biasing potentials within simulations as shown in the presented umbrella sampling approach to examine the barrier height. We conclude that extra care needs to be taken by molecular modelers to accurately describe cis/trans conformational states in urea fragments and thus to avoid major flaws in sytem energetics. A variety of tools including quantum-mechanical calculations, knowledge-based approaches as well as biased simulation techniques might be helpful to face this additional challenge.

## Electronic supplementary material

Below is the link to the electronic supplementary material.

Supplementary material 1 (PDF 2092 kb)
