# Supplementary material for: Kinetic barriers in the isomerization of substituted ureas: implications for computer-aided drug design
Source: J Comput Aided Mol Des. 2016 Jun 7;30:391–400. doi: 10.1007/s10822-016-9913-4 (PMC4912590; doi:10.1007/s10822-016-9913-4)

Supporting Information:

Kinetic Barriers in the Isomerization of Substituted Ureas: Implications for Computer-Aided Drug Design

Johannes R. Loeffler, Emanuel S. R. Ehmki, Julian E. Fuchs*, Klaus R. Liedl

Institute of General, Inorganic and Theoretical Chemistry, Faculty of Chemistry and Pharmacy, University of Innsbruck, Innrain 82, A-6020 Innsbruck, Austria

*E-mail: julian.fuchs@uibk.ac.at

Supporting Figure 1:

Chemical structure of ligands used in thermodynamics integration calculations. A) Template ligand with CF_3 group, B) target ligand with SCF_3 substitution, C) a conformational switch to the cis/trans urea conformation in the target ligand is simulated.

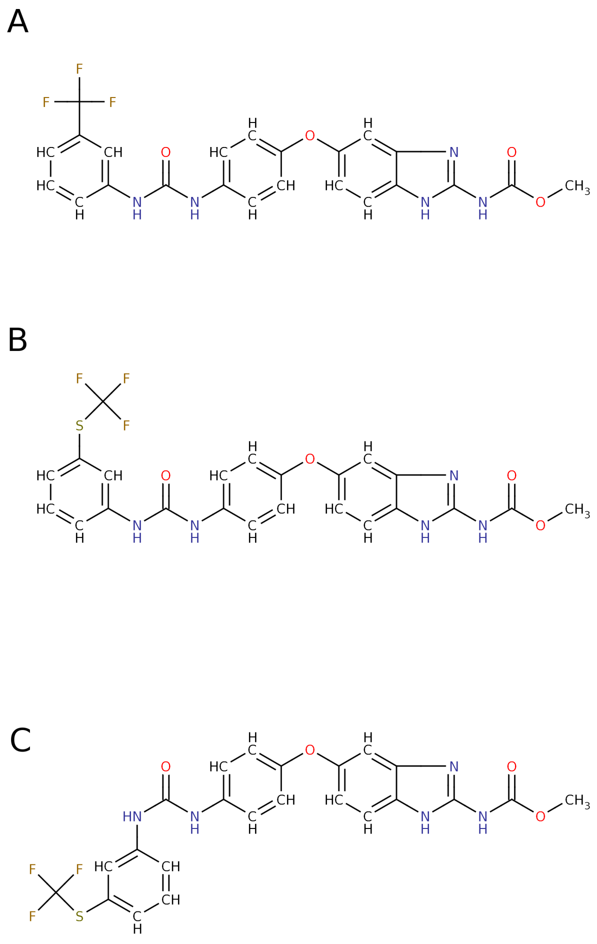

Supporting Figure 2:

Free energy profiles recovered from thermodynamics integration calculations. A) Profile for the ligand transformation in trans/trans conformation in solvent, B) profile for the transformation as protein-ligand complex with the VEGFR-2 tyrosine kinase in trans/trans conformation, C) profile for the transformation from the trans/trans template ligand to the cis/trans target ligand in solvent, D) profile for the same transformation in the binding pocket of VEGFR-2 tyrosine kinase, E) profile for the ligand inversion of the template ligand from trans/trans to cis/trans, F) profile for the reverse transformation from cis/trans to trans/trans.

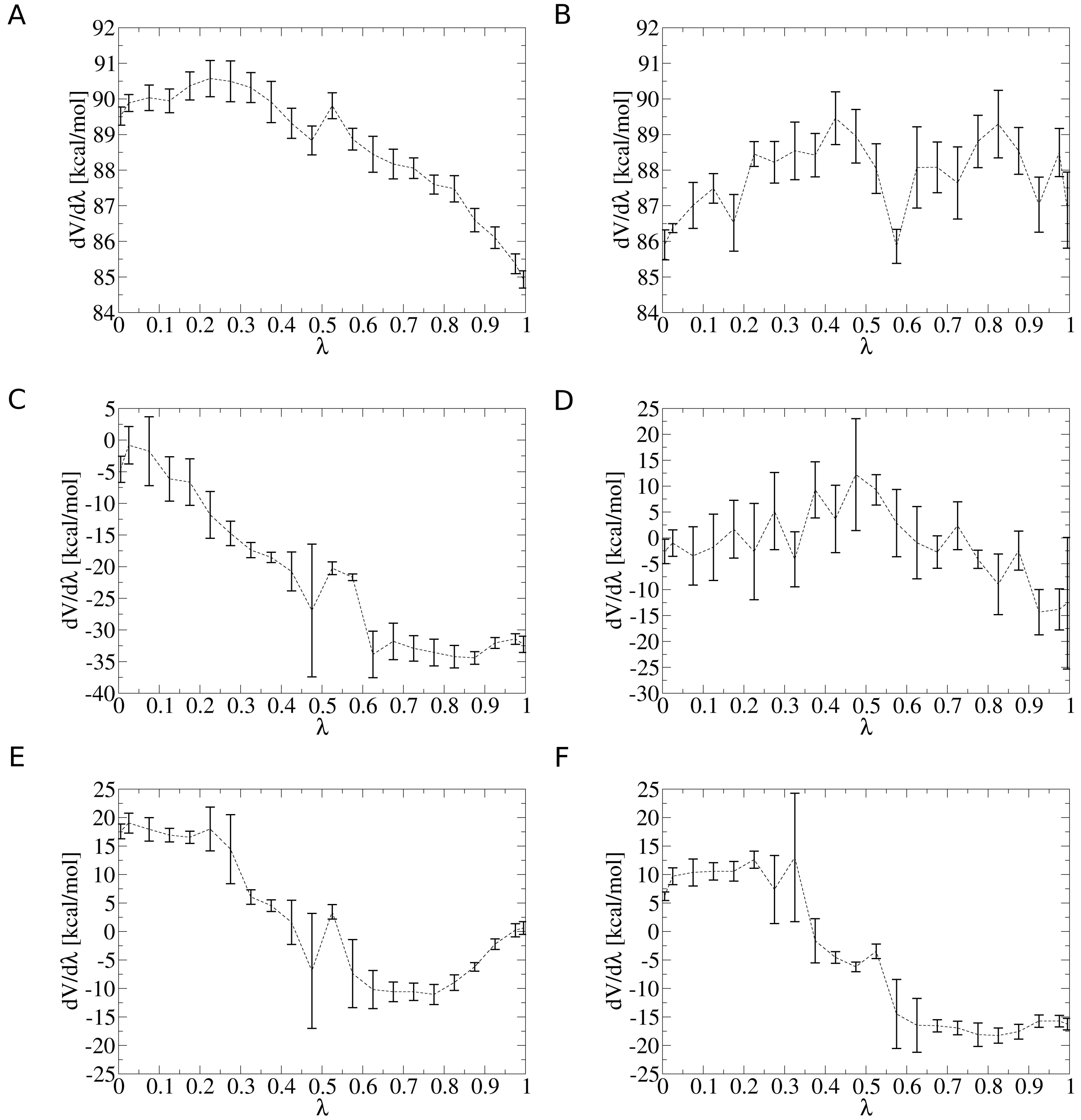

Supplement: Supplementary file 1 — Supplementary material 1 (PDF 2092 kb) [file 10822_2016_9913_MOESM1_ESM.pdf]
